# The effect of low and high-intensity cycling in diesel exhaust on flow-mediated dilation, circulating NO_x_, endothelin-1 and blood pressure

**DOI:** 10.1371/journal.pone.0192419

**Published:** 2018-02-21

**Authors:** Luisa V. Giles, Scott J. Tebbutt, Christopher Carlsten, Michael S. Koehle

**Affiliations:** 1 School of Kinesiology, University of British Columbia, Vancouver, British Columbia, Canada; 2 Department of Medicine, University of British Columbia, Vancouver, British Columbia, Canada; 3 Centre for Heart Lung Innovation, University of British Columbia and St. Paul’s Hospital, Vancouver, British Columbia, Canada; 4 School of Population and Public Health, University of British Columbia, Vancouver, British Columbia, Canada; 5 Division of Sports Medicine, University of British Columbia, Vancouver, British Columbia, Canada; Université libre de Bruxelles, BELGIUM

## Abstract

**Introduction:**

Exposure to air pollution impairs aspects of endothelial function such as flow-mediated dilation (FMD). Outdoor exercisers are frequently exposed to air pollution, but how exercising in air pollution affects endothelial function and how these effects are modified by exercise intensity are poorly understood.

**Objectives:**

Therefore, the purpose of this study was to determine the effects of low-intensity and high-intensity cycling with diesel exhaust (DE) exposure on FMD, blood pressure, plasma nitrite and nitrate (NOx) and endothelin-1.

**Methods:**

Eighteen males performed 30-minute trials of low or high-intensity cycling (30% and 60% of power at VO_2peak_) or a resting control condition. For each subject, each trial was performed once while breathing filtered air (FA) and once while breathing DE (300ug/m^3^ of PM_2.5_, six trials in total). Preceding exposure, immediately post-exposure, 1 hour and 2 hours post-exposure, FMD, blood pressure and plasma endothelin-1 and NOx concentrations were measured. Data were analyzed using repeated-measures ANOVA and linear mixed model.

**Results:**

Following exercise in DE, plasma NOx significantly increased and was significantly greater than FA (p<0.05). Two hours following DE exposure, endothelin-1 was significantly less than FA (p = 0.037) but exercise intensity did not modify this response. DE exposure did not affect FMD or blood pressure.

**Conclusion:**

Our results suggest that exercising in DE did not adversely affect plasma NO_X_, endothelin-1, FMD and blood pressure. Therefore, recommendations for healthy individuals to moderate or avoid exercise during bouts of high pollution appear to have no acute protective effect.

## Introduction

The endothelium is a thin layer of cells located on the inner lumen of the blood vessel which sense mechanical and chemical stimuli and respond by releasing substances that regulate vascular tone, cell adhesion, thrombosis, smooth cell proliferation and inflammation.[1] Endothelial dysfunction is characterized by impaired vasodilation, a pro-inflammatory state, and pro-thrombotic tendencies [2] and is considered a key event in the development of atherosclerosis.[3]

Compared to active individuals, sedentary individuals have impaired endothelial function as demonstrated by attenuated vasodilation in response to mechanical stimuli,[4] and improvements in endothelial function occur with regular exercise.[5] Conversely, in healthy individuals and in those with cardiovascular disease, exposure to air pollution containing PM impairs endothelial function [6] and specifically diesel exhaust (DE) exposure impairs vascular and fibrinolytic function [7] and components of DE (such as PM) cause vasoconstriction and endothelial dysfunction.[6]

The effects of exercise on the cardiovascular system are clear; specifically, the abovementioned improvement in endothelial function with regular exercise is a key mechanism in the reduction of cardiovascular disease risk.[8] However, during exercise, factors such as greater minute ventilation, higher particle deposition fraction, total particle deposition, and oral breathing increase the dose of air pollution,[9,10] which may result in greater air pollution-mediated adverse effects than during rest. Furthermore, with increasing exercise intensity, the dose of air pollution should theoretically be greater, which could then increase the magnitude of physiological and health effects of air pollution beyond that during rest or lower intensity exercise. However, the limited research on how PM or DE exposure during exercise acutely affect endothelial function is conflicting.[11–14] For example, in healthy individuals, some research suggests that acute exposure to high levels of PM_1_ (gasoline engine) during exercise impairs vascular function as measured by FMD [11,12,15] and increases pulmonary artery pressure [11]. In contrast, acute exposure to high levels of PM_2.5_ with exercise does not cause microvascular dysfunction [16,17] despite the dysfunction being present with rest [17], implying that exercise may offset the adverse effects of PM. Of the studies assessing the effect of exercising in air pollution on endothelial/vascular function no studies address how exercise intensity could alter the physiological response to air pollution.

Understanding how a key mechanism in cardiovascular health, such as endothelial function, is affected by DE exposure during exercise would provide greater insight into the interaction between air pollution, exercise, and health. A better insight into this relationship would also allow us to advise individuals about how to modify exercise routines during bouts of high air pollution. To this end, the purpose of this study was to determine the acute endothelial responses to low- and high-intensity cycling with DE exposure. We hypothesized that DE exposure would impair the normal endothelial response to exercise and rest and any physiological effects due to DE would be magnified as exercise intensity increases.

## Materials and methods

Data collection for this study occurred as part of a larger study looking at interactions between exercise and air pollution. Overall methodology is explained in detail in another publication,[18] but briefly: eighteen recreationally active male participants attended the laboratory on seven occasions. The initial visit served for familiarization and maximal exercise testing. On the remaining testing days, participants performed 30-min trials of low-intensity cycling, high-intensity cycling, or rest. Each intensity (including rest) was performed once in filtered air (FA) and once in DE containing 300 μg/m^3^ of PM_2.5_, for a total of six trials, each of which was separated by a 7-day period and occurred at the same time of day. Exercise intensity and the exposure (FA and DE) were randomized with both the participant and the researcher blinded to the exposure condition. Prior to all visits, participants were asked to refrain from food high in nitrites and nitrates for 48 h, exhaustive exercise and alcohol for 24 h, caffeine for 6 h, and food or non-water beverages for 2 h. Participants were also asked to maintain the same pre-test routine including the same mode of travel to the laboratory and pre-test meal, and were asked to avoid vitamin supplementation for the duration of the study.

The Clinical Research Ethics Board of the University of British Columbia approved this study. Participants had an orientation session and a reflection period before signing the written informed consent. The sample size was calculated based on a minimal detectable difference in FMD of 1.59%, using an effect size of 0.32, a power of 0.8, and an alpha of 0.05,[11] and a minimal detectable difference in endothelin-1 of 0.27 pg/ml, using an effect size of 0.59, a power of 0.8, and an alpha of 0.05.[19]

### Exercise apparatus

Exercise tests were performed using a Velotron Dynafit Pro cycle ergometer (Racermate Inc, Seattle, WA, USA). During trials, participants breathed through a facemask (7450 Series, Hans Rudolph Inc, Kansas City, MO, USA) attached to a low-resistance, non-rebreathing valve (NRB 2700, Hans Rudolph Inc, Kansas City, MO, USA) to collect respiratory and metabolic data that have been previously reported.[18] Participants remained outside the environmental booth but were connected to the booth via 3.2 cm diameter hoses (made from a high performance ethylene-vinyl acetate copolymer) at both the inspired and expired sides of the non-rebreathing valve.

### Introductory session (Day 1)

Day 1 consisted of familiarization with all study procedures and performance of a maximal exercise test on a cycle ergometer. For the maximal exercise test, the cycling work rate started at 100 W and increased by 0.5 W/s until volitional exhaustion. Peak power was taken as the power output before cycling cadence dropped below 60 revolutions per min. ${\dot{V}O}_{2Peak}$ values were taken as the highest 10 s average. To exclude those subjects with possible exercised-induced bronchoconstriction, any individual with a post-exercise decrease in forced expiratory volume in 1 second by 10% or greater was excluded from the study.

### Testing days (Days 2–7)

Testing Days 2–7 consisted of 30 min trials of cycling or 30 min of rest. Thirty minutes of exercise/exposure was chosen, as it was the longest duration that participants could cycle at the highest intensity, additionally it was chosen to represent the average duration of a cycle commute.[20] Work rates on cycling days were based on the peak power achieved during the maximal exercise test. Low-intensity cycling was set at 30% of the power at. ${\dot{V}O}_{2Peak}$ (mean (sd): 96.1 (17.7) W) and high-intensity cycling was set at 60% of power at. ${\dot{V}O}_{2Peak}$ (192.2 (35.3) W). Control exposures involved sitting for the same period of time (30 min), but without performing exercise. Prior to, immediately post, 1 h, and 2 h post-exposure endothelial function by flow-mediated dilation (FMD), blood pressure, plasma endothelin-1 and the sum of plasma nitrite and nitrate (plasma NO_x_) were measured.

### Endothelial function

Endothelial function was measured by FMD; this technique assesses the change in diameter of the brachial artery following occlusion of the vessel with a pneumatic cuff. Prior to each sampling test, participants rested supine for 20 min. During the test, each participant’s left arm was extended and supported at an abduction angle of ~80° from the torso. For assessment of the FMD response, a blood pressure cuff was positioned on the imaged arm, distal to the olecranon process to provide a stimulus of forearm ischemia. The brachial artery was imaged in the distal third of the upper arm; the site of measurement as well as transducer angle was the same for each person for all trials. Following a 20-min resting period, the brachial artery diameter was measured at baseline for one min. Subsequently, the occlusion cuff was inflated to >200 mmHg for five min. Brachial artery diameter recordings were made at least 30 s before cuff deflation and continued for at least three min after deflation.

#### Endothelial function apparatus

A Logiq I portable ultrasound scanner in 2D (General Electric Inc., Fairfield CT, USA), with a vascular transducer, was used to obtain continuous images. These images were then captured as a video using a commercially available frame grabber (VGA2USB LR frame grabber, Epiphan Systems, Ottawa, Ontario) at 10 frames per second. Brachial artery diameters were analyzed from the video continuously using an edge detection and wall-tracking software (Medical Image Applications, Vascular Research Tolls 5, Coralville, IA). One individual who was blinded to the exposure condition performed scans and subsequent analysis. Baseline brachial artery diameter coefficient of variation was 4 (1.9)%, which is similar to other studies. [21]

#### Endothelial function data processing

Brachial artery diameter was measured in 17 of 18 participants, due to an inability to acquire adequate images in one participant. Data acquired during imaging included pre-occlusion brachial artery diameter, peak post-occlusion brachial artery diameter, FMD, and time to peak dilation (TTP). Pre-occlusion artery diameter was determined as the mean of one min prior to cuff inflation. Peak post-occlusion artery diameter was automatically identified as the maximum median within a 30-frame bracket of data (3 seconds). Each bracket of data included a 20% overlap with the previous bracket. The maximum value of all the calculated median values was automatically detected and chosen to represent the peak of the diameter curve. Flow-mediated dilation was calculated as the percentage rise of this peak diameter from the preceding baseline diameter. One measure of brachial artery diameter was not used due to a technical error during the measurement; to prevent complete exclusion of this participant, the missing value was imputed using regression.[22]

### Plasma endothelin-1 and NOx

Blood samples were taken from the right antecubital fossa with a 21-gauge needle. All blood samples were immediately centrifuged at 1500 *g* for 20 min to separate plasma from formed elements. Plasma was extracted, frozen, and stored at -80°C until assayed. Plasma concentrations of endothelin-1 were determined in duplicate using commercially available enzyme-linked immunosorbent assay (ELISA) kits (Endothelin-1 Immunoassay Quantikine ELISA, R&D Systems, MN, USA) and according to the procedures outlined by the manufacturer. Plasma levels of endothelin-1 were measured using a Versa Max microplate reader (Molecular Devices Corporation, CA, USA). The intra assay coefficient of variation for endothelin-1 was 3.5%.

Nitric oxide has a very short half-life; therefore, end products of nitric oxide such as nitrate and nitrite are used as indicators of NO production, and the sum of plasma nitrite and nitrate is referred to as plasma NOx. Plasma nitrite and nitrate levels were determined in duplicate using a commercially available assay kit (total nitric oxide and nitrate/nitrite assay, R&D Systems MN, USA) according to the procedures of the manufacturer. Plasma levels of nitrite and nitrate were measured using a BioTek 96-well plate reader (BioTek Instruments, Winooski, VT, US). The intra assay coefficient of variation for nitrite and nitrate assays within the current study was 6.2%. Of the 432 blood samples taken two samples were unable to be collected. To prevent complete exclusion of those subjects with missing measurements and based on the recommendations of a statistician, the missing values were replaced using regression imputation.[22]

Levels of endothelin-1 and plasma NOx were adjusted for changes in plasma volume from baseline. The estimated post-exercise concentration of markers due to plasma volume changes alone was estimated using the following equation:[23]

$${Concentration}_{ESTIMATED=\frac{{Hct}_{POST}X(100-{Hct}_{PRE})}{{Hct}_{PRE}X(100-{Hct}_{POST})}X{Concentration}_{PRE}}$$

where *Hct* represents hematocrit. The adjusted concentration was then calculated by subtracting the estimated concentration due to plasma volume changes from the pre-concentration; this difference was then added to the measured concentration within the plasma and is detailed in the following equation:

$${Concentration}_{ADJUSTED}=({Concentration}_{PRE}-{Concentration}_{ESTIMATED})+{Concetration}_{MEASURED}$$

### Blood pressure

Blood pressure was measured following 20 minutes of supine rest. Systolic blood pressure (SBP) and diastolic blood pressure (DBP) were measured in triplicate using an automated device (BPA-060-0CA, HoMedics, Commerce Township, MI, USA) with at least one min between measures and the average of the final two measurements was used. Mean arterial pressure (MAP) was estimated as ((2•DBP)+SBP)/3.

### Exposure setup

All exposures were performed using an environmental exposure booth that is explained in detail elsewhere,[24] but was modified only in that load was constant at 2.5kW. For DE exposures, participants were exposed to calibrated, aged, and diluted DE containing 300μg/m^3^ of PM_2.5_. In-booth PM mass concentration measurements were made using a Tapered Element Oscillating Microbalance (TEOM; Model 1400a, Rupprecht & Pattashnick, Albany, NY, USA) using 10 min averages. A TSI Scanning Mobility Particle Scanner (Model 3936, TSI, Shoreview, MN, USA) classified the particle size distribution between 2.5 nm and 1000 nm. Thermo Model 48C and Model 42C analyzers (Thermo Fisher Scientific, Mississauga, ON, Canada) were used to measure and record carbon monoxide and oxides of nitrogen concentration levels in the exposure booth, respectively. A GrayWolf TG-503 probe was placed within the exposure booth and used to measure the total volatile organic compounds (TVOC), relative humidity, and temperature in real-time. For FA exposures, participants were exposed to compressed, HEPA-filtered air.

### Statistical analysis

Statistical analyses were completed using SPSS software (SPSS Inc, version 20, Chicago, IL) and analyses were chosen through consultation with a statistician. Data were analyzed using a 2 (exposure: FA vs. DE) x 3 (intensity: rest, low-intensity, high-intensity) x 4 (time: pre, post, 1 h, 2 h) repeated measures ANOVA. For all repeated measures ANOVA the Huynh-Feldt adjustment was used to correct for violations of sphericity. Main or interaction effects were further analyzed using pair-wise comparisons and significance was adjusted to account for multiple comparisons using the Sidak adjustment. Analysis (results detailed below) revealed that exercise affects pre-occlusion baseline diameter which may influence the degree of FMD.[25] Therefore FMD was subsequently analysed using a linear mixed model similar to previously described analysis.[26] Specifically, pre-occlusion artery diameter and peak diameter were logarithmically transformed (LogPre-occlusion and LogPeak) and the change in diameter was calculated on the logarithmic scale (ΔLogMM). In the mixed model, the ΔLogMM was entered as the dependent variable, the exposure condition, exercise intensity and time were entered as fixed factors, and LogPre-occlusion diameter as a random factor.[27] Covariate adjusted means for ΔLogMM were back-transformed and then converted to a corrected FMD by subtracting 1 from the back-transformed values and multiplying by 100.[26] All means related to the linear mixed model are presented as corrected FMD values and the statistical results presented are those obtained using the ΔLogMM in the linear mixed model. All means are reported with standard deviations in parentheses and data are considered statistically significant if p<0.05.

## Results

Eighteen recreationally active males (age 24.5 (6.2) yr (mean (sd)); height: 1.78 (0.08) m; body mass: 74.2 (10.5) kg) had a mean. ${\dot{V}O}_{2Peak}$of 55.0 (9.1) mL•kg^-1^•min^-1^, a mean maximum power output of 320.4 (58.9) W, and a mean maximum heart rate of 182.1 (12.7) bpm.

Baseline levels of all outcome variables were not significantly different across the six test days (p>0.05). All participants performed all six trials, although three participants were unable to finish the high-intensity trial in DE due to volitional exhaustion. In individuals who were unable to finish the first high-intensity trial, the second high-intensity exercise trial was designed to mimic the first; therefore, the duration in trial two was matched to that of the first trial. Mean (SD) PM_2.5_ during DE and FA exposures were 302.1 (6.50) and 9.30 (6.20) μg/m^3^ respectively. Mean particle number concentration (PNC) during DE and FA exposures were 0.14 x 10^4^ and 61.60 x 10^4^ (#/cm^3^). Median particle diameter during DE and FA exposures was 59.4 (1.80) and 87.70 (0.60) nm. Mean NO_2_ during DE and FA exposures was 0.04 (0.04) and 0.58 (0.15) ppm. Mean NO during DE and FA exposures was 0.02 (0.02) and 7.00 (0.09) ppm. Mean carbon monoxide during DE and FA exposures was 3.00 (0.40) and 13.9 (2.10) ppm. Total VOC’s during DE and FA exposures was 106 (62.8) and 1310 (226.3) ppb.

### Endothelial function

**Fig 1:**
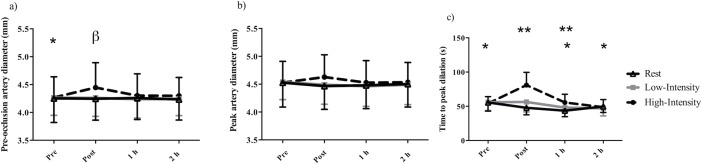
Mean (a) pre-occlusion artery diameter, (b) peak artery diameter, (c) time to peak dilation in 17 recreationally active males prior to and following rest, low-intensity, or high-intensity cycling. Error bars represent SD. * = significantly less than post for high-intensity only. ** = significant difference between high-intensity compared to low-intensity and rest at the same time point. β = significant difference between high-intensity compared rest at the same time point.

Mean baseline pre-occlusion diameter, peak artery diameter, FMD, and TTP were 4.26 (0.35) mm, 4.53 (0.59) mm, 6.5 (1.6) %, and 52.2 (9.3) s respectively. The repeated measures ANOVA indicated that there was a significant two-way interaction (intensity-by-time) for pre-occlusion artery diameter (p = 0.003, Fig 1A), peak artery diameter (p = 0.005, Fig 1B), and TTP (p<0.001, Fig 1C), which all increased following high-intensity exercise. Immediately following high-intensity exercise, pre-occlusion artery diameter was significantly greater than pre-exercise (p = 0.01, 4.45 (0.45) mm vs. 4.27 (0.37) mm). Pre-occlusion artery diameter was significantly greater in high-intensity exercise compared to rest (p = 0.04, 4.45 (0.45) mm vs. 4.25 (0.39) mm,). *Post hoc* comparisons for peak artery diameter did not reveal significant differences. Compared to pre exposure/exercise measures TTP significantly decreased 1 h follow rest (p = 0.01, 55.53 (12.69) s vs. 43.58 (8.80) s). In contrast, TTP was significantly greater immediately following high-intensity cycling (80.96 (18.66) s) compared to pre-exercise (p<0.001, 54.19 (9.91) s), and 1 h (p<0.001, 55.56 (12.49) s) and 2 h post exercise (p<0.001, 48.74 (11.12) s). TTP was significantly greater immediately post high-intensity cycling (80.96 (18.66) s) compared to low-intensity (p<0.001, 56.29 (14.58)) and rest (p<0.001, 47.83 (10.22) s) and 1 h post high-intensity cycling (55.56 (12.09) s) compared to low-intensity (p = 0.04, vs. 48.10 (6.40) s) and rest (p = 0.03,43.58 (8.80) s).

The repeated measures ANOVA did not reveal any significant interaction effects (exposure-by-intensity-by-time: F = 1.021, p>0.05; exposure-by-intensity: F = 3.362, p>0.05; exposure-by-time F = 0.477, p>0.05; intensity-by-time: F = 0.702, p>0.05) but revealed a main effect of time for FMD (p = 0.01) and *post hoc* analysis revealed that immediately following exercise/exposure, FMD was significantly less than pre exercise/exposure (p = 0.003, 6.40 (1.61) % vs. 5.00 (1.75) %).

A linear mixed model analysis revealed that time (F = 7.482, p<0.001) had a significant effect on ΔLogMM. Specifically, prior to exercise/exposure, ΔLogMM was significantly greater than immediately following (p<0.001, Estimate: -0.011 (0.01)) and 1 hr post (p = 0.001, Estimate: -0.010 (0.01)). A summary of uncorrected and corrected FMD values can found in Table 1.

**Table 1:** The main effects of time, exposure condition and intensity on FMD (Mean (SD)) in 17 recreationally active males.

	Uncorrected FMD	Corrected FMD
	Mean (SD)	
*Prior to and following exercise or rest*		
Pre	6.40 (1.61)	5.30 (1.16)
Post	5.00 (1.75)*	4.08 (1.10)*
1 h post	5.32 (1.74)	4.31 (1.15)*
2 h post	5.88 (1.33)	4.85 (1.14)**
*Diesel exhaust and filtered air*		
Filtered Air	5.65 (1.39)	4.62 (0.99)
Diesel	5.65 (1.30)	4.65 (1.00)
*Rest*, *low and high intensity exercise*		
Rest	5.84 (1.31)	4.75 (1.12)
Low Intensity	5.79 (1.58)	4.69 (1.10)
High-Intensity	5.32 (1.55)	4.46 (1.05)

* significantly less than pre-exercise in the corresponding column, p<0.05

** significantly less than post, p< 0.05

Uncorrected data is obtained from estimated means from the repeated measures ANOVA. All corrected data are calculated through back transformation of ΔLogMM from the linear mixed model. The statistical results presented are those obtained using the ΔLogMM in the linear mixed model.

### Plasma NO_x_

**Fig 2:**
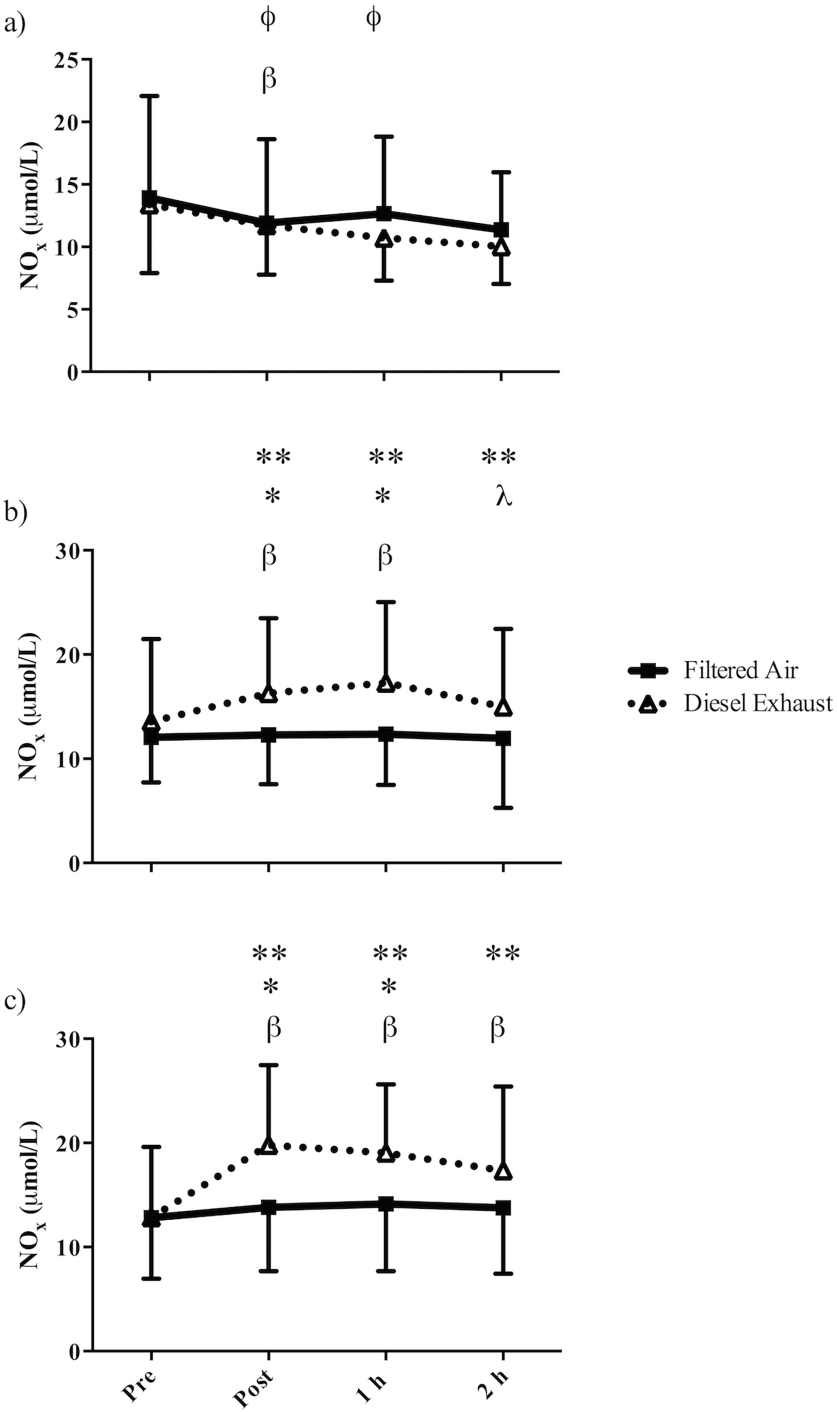
Mean plasma NO_x_ in 18 recreationally active males prior to and following 30-min of (a) rest, (b) low-intensity cycling, or (c) high-intensity cycling in filtered air or diesel exhaust. Error bars represent SD. β = significantly different from pre-exercise in the corresponding exposure, in (a) FA is only significant, in (b) and (c) DE is only significant; ϕ = significantly different from 2h occurs in DE only; * = significantly greater than FA at the corresponding time point; ** = significantly greater than rest at the corresponding time point (comparing DE only); λ = significantly different from 1 h occurs in DE only.

There was a significant three-way interaction (exposure-by-intensity-by-time) for plasma NOx (Fig 2, p = 0.006). *Post hoc* analysis showed that following low- and high-intensity exercise in DE but not FA, plasma NOx levels were significantly higher than pre-exercise values (Low-intensity: pre vs. post p = 0.013, pre vs. 1 h post p<0.001; high-intensity: pre vs. post p<0.001, pre vs. 1 h post p<0.001, pre vs. 2 h post p = 0.001). Following low- and high-intensity exercise, plasma NOx values were significantly higher in DE compared to FA (Low-intensity: immediately post p = 0.02, 1 h post p = 0.07; high-intensity: immediately post p = 0.02, 1 h post p = 0.04). Plasma NOx was significantly greater following low- and high-intensity exercise in DE compared to rest (Immediately post: rest vs. low-intensity p = 0.024, rest vs. high-intensity p<0.001; 1 h post: rest vs. low-intensity p = 0.002, rest vs. high-intensity p<0.001; 2 h post: rest vs. low-intensity p = 0.012, rest vs. high-intensity p<0.001). Immediately post-rest reduction in plasma NOx in FA (p = 0.005).

### Endothelin-1

**Fig 3:**
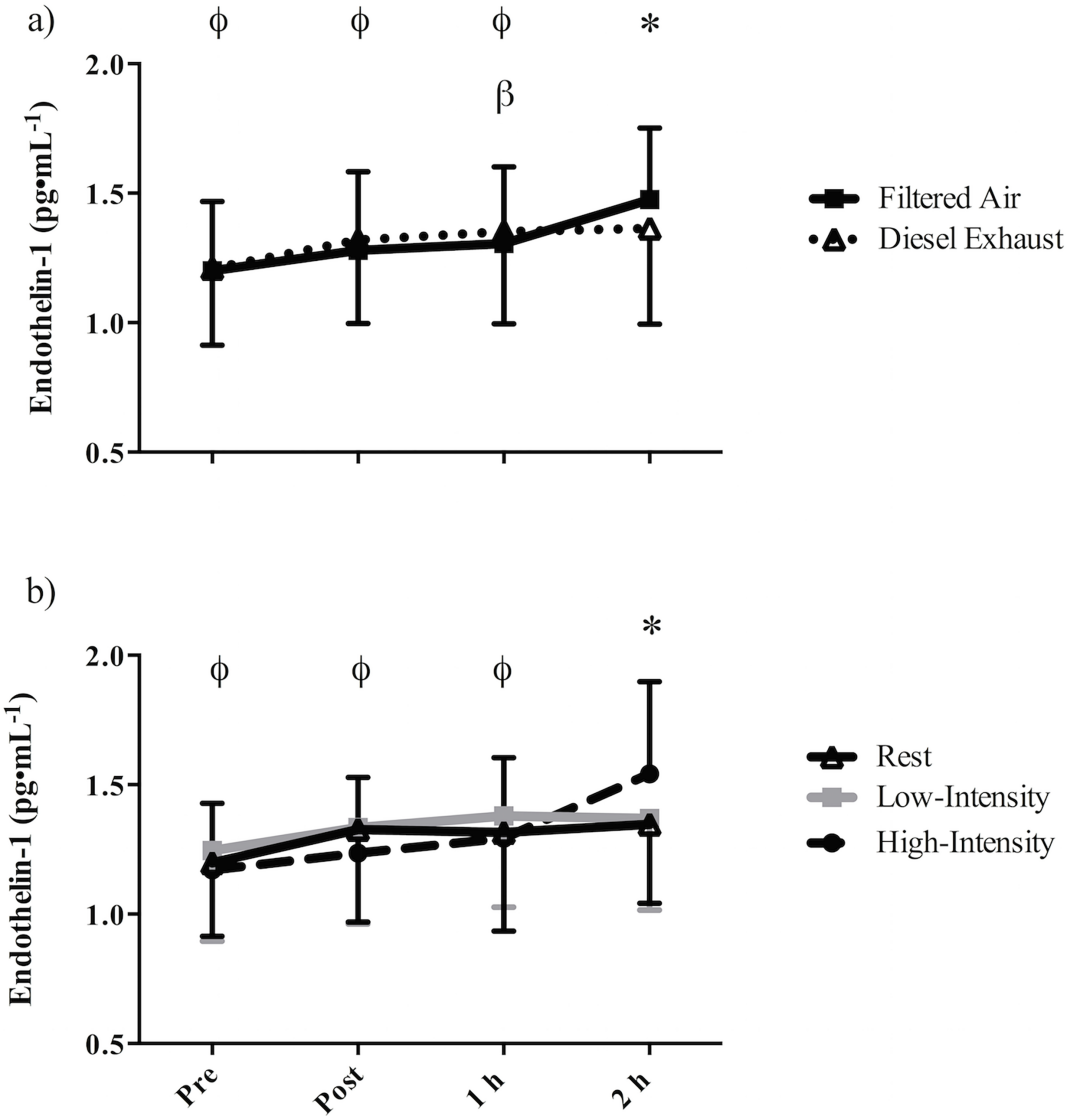
Mean plasma endothelin-1 in 18 recreationally active males pre, post, 1 h- and 2 h- post (a) 30-min of exposure to filtered air or diesel exhaust (exposure-by-time interaction), or (b) 30-min of rest, low-intensity cycling, or high-intensity cycling (intensity-by-time interaction). Error bars represent SD. β = significantly greater than pre-exercise (DE only); * = (a) DE significantly less than FA, (b) Rest and low-intensity significantly less than high-intensity at the corresponding time point; ϕ = (a) significantly less than 2 h post occurs in FA only, (b) significantly less than 2 h post occurs in high-intensity only.

There was a significant exposure-by-time interaction for plasma endothelin-1 (p = 0.003; Fig 3A). In both FA and DE, endothelin-1 levels increased over time; specifically, 2h following exposure to FA, plasma endothelin-1 (1.48 (0.28) pg•ml^-1^) was significantly greater than pre exposure (p<0.001, 1.20 (0.27) pg•ml^-1^), post exposure (p = 0.003, 1.28 (0.30) pg•ml^-1^) and 1 h post exposure (p = 0.005, 1.31 (0.30) pg•ml^-1^). One hour following exposure to DE, endothelin-1 (1.35 (0.36) pg•ml^-1^) was significantly greater than pre exposure (p = 0.023, 1.21 (0.30) pg•ml^-1^). Two hours following exposure to DE, endothelin-1 (1.36 (0.37) pg•ml^-1^) was significantly less than following FA (p = 0.037, 1.48 (0.28) pg•ml^-1^).

There was also a significant intensity-by-time interaction (p = 0.001, Fig 3B) for endothelin-1. Two hours following high-intensity cycling plasma endothelin-1 levels (1.54 (0.36) pg•ml^-1^) were significantly greater than pre exercise (p = 0.001, 1.17 (0.26) pg/ml), post exercise (p = 0.005, 1.24 (0.29) pg•ml^-1^), and 1 h post exercise (p = 0.024, 1.29 (0.31) pg•ml^-1^). Endothelin-1 was significantly greater 2 h post high-intensity exercise compared to 2 h following low-intensity exercise (p = 0.003, 1.37 (0.35) pg•ml^-1^) and rest (p = 0.035,1.35 (0.31) pg•ml^-1^)

### Blood pressure

There was a main effect of intensity on MAP (p = 0.03, 86.79 (5.72) mmHg vs. 86.06 (4.65) mmHg vs. 85.27 (4.63) mmHg, for rest, low-intensity, and high-intensity respectively); however, *post hoc* analysis did not reveal significant differences. There was a significant intensity-by-time interaction for SBP (p = 0.014); 1 h following rest SBP (118.50 (8.27) mmHg) was significantly higher than 1 h following low-intensity (p = 0.005, 114.60 (6.33) mmHg) and 1 h following high intensity cycling (p = 0.036, 114.22 (6.11) mmHg). Additionally, 2 h following high-intensity cycling, SBP (114.85 (6.73) mmHg) was significantly lower than following rest (p<0.001,119.51 (8.84) mmHg) or low-intensity cycling (p = 0.001, 117.93 (7.87) mmHg).

## Discussion

This is the first study to examine how parameters of vascular function respond to exercise of varying intensities in DE. We found that following exercise in DE, plasma NOx was significantly increased, and immediately post and 1 h post-exercise, plasma NOx levels were significantly higher in DE compared to FA. Additionally, 2 h following 30 min of exposure to DE, endothelin-1 was significantly lower compared to FA. Irrespective of environmental exposure, we found an effect of exercise intensity on endothelin-1, pre-occlusion artery diameter and TTP, which all increased following high-intensity exercise, and an effect of time on FMD which was lower immediately following and 1 hr following exercise/ exposure compared to prior to exercise/exposure.

Nitric oxide plays an important role in the maintenance of vascular tone and endothelial function and therefore FMD.[28] In the present study, following low- and high-intensity exercise in DE, plasma NOx (the sum of plasma nitrite and nitrate) levels increased, but as there were no differences between high-intensity and low-intensity exercise this does not support our hypothesis that exercise intensity magnified any DE related physiological effects. The increase in plasma NOx during low- and high-intensity exercise in DE was significantly greater than immediately post and 1 hour post-exercise in FA. This increase is in accordance with other studies;[29,30] but given the multiple post-exposure time points, this study provides a greater resolution of the response of NO end-products following DE exposure over time. Some authors have suggested that the elevated levels of these NO end-products following DE exposure may represent an up-regulation of vascular NO generation.[30] The up-regulation may have occurred to compensate for the NO that is consumed during a state of increased oxidative stress that results from exposure to air pollution as well as in the maintenance of vascular tone and blood pressure.[30] In contrast, others suggested that the higher NO end-products are simply related to higher absorption of oxides of nitrogen present in the DE,[29] which is supported by studies showing that inhalation of 80 ppm of NO increased blood nitrate levels fourfold.[31] In the present study, DE used in the exposure contained 7 ppm of NO, which is more than a magnitude lower than NO exposure studies (80 ppm) [31] and may explain why plasma NOx levels did not increase during rest. The higher ventilation along with the resulting four- and sevenfold increase in the inhaled dose of NO during low- and high-intensity exercise may account for the increase in plasma NOx seen with exercise in DE.

The higher circulating plasma NOx following exercise in DE may not necessarily be beneficial, as DE can result in the production of superoxide,[32] which may then combine with NO to form powerful oxidants such as peroxynitrite. Additionally, in the presence of exogenous NO or DE, *de novo* synthesis of NO is lowered through a down-regulation of endothelial nitric oxide synthase (eNOS) [33,34] or uncoupling of eNOS.[35,36] The term eNOS uncoupling is used to describe when eNOS is altered and produces reactive oxygen species such as superoxide instead of NO.[37] Therefore, with DE exposure, the uncoupling of eNOS could result in the production of reactive oxygen or reactive nitrogen species, rather than the production of NO.[28,38] The higher levels of circulating NOx in the current study may then combine with reactive oxygen species via redox reactions to form peroxynitrite.[39,40] The interaction of NO with reactive oxygen species such as superoxide could therefore result in oxidative stress. A study by Nurkiewicz et al., exposing rats to inhaled titanium dioxide nanoparticles (used as a surrogate for environmental PM) confirmed this interaction, which led to oxidative stress in the microvasculature.[28] Despite the study being conducted in rats, it is possible that the same process occurs in humans; therefore, in the current study, the elevated circulating NOx due to exogenous NO exposure, as well as a potentially greater superoxide production due to DE, could cause these molecules to combine which may result in a cascade of oxidative stress. However, as this study did not directly measure products of oxidative stress it is unclear if the excess NO would result in oxidative stress.

Endothelin-1 is a vasoconstrictor that opposes the effects on nitric oxide and thus plays a role in the maintenance of vascular tone.[41] An exposure-by-time interaction showed that at 2 h post-exposure, endothelin-1 was significantly less in DE compared to FA. The decrease in endothelin-1 is in contrast to others who found that 2 h of exposure to DE during rest either increased [19] or did not affect endothelin-1 [42] and 1 hour exposure during intermittent exercise [7] also did not affect endothelin-1. It is possible that the greater endothelin-1 2 h following FA (exposure-by-time interaction) is driven by high-intensity exercise (represented by an intensity-by-time interaction shown in Fig 3B). Despite endothelin-1 levels 2 h post high-intensity exercise being greater by 0.2 pg/ml in FA (1.64 vs. 1.44 pg/ml, *post-hoc* comparison p = 0.042), as this study did not find a significant three-way interaction effect (exposure-by-intensity-by-time) these data cannot support the hypothesis that high-intensity exercise in FA significantly increases endothelin-1 compared to DE. It is unclear why endothelin-1 would be lower in DE, but it is possible that if the exercise challenge in this study were shorter and more severe that a post-exercise increase in endothelin might have been observed. Alternatively, as plasma endothelin-1 may represent the balance of spillover from local release and elimination/use, the lower concentration of endothelin-1 in DE may represent a greater elimination/use or binding of endothelin-1 to its receptors, which may ultimately result in vasoconstriction.

Endothelial function plays a role in the development of atherosclerosis [3] and predicts future cardiovascular events in those with cardiovascular disease [43]. The endothelium typically responds to short-term increases in shear stress by increasing synthesis of NO and other vasodilators that dilate the artery wall [44]. Flow-mediated dilation is a technique used to assess NO-mediated dilation and endothelial function.

The lack of effect of DE exposure on FMD during rest could be related to the fitness level of participants. As an example, Brauner et al [16] did not find that exposure to particulate air pollution influenced vascular dysfunction in healthy individuals. Similarly, in mice with slight atherosclerosis diesel exhaust particle exposure resulted in endothelial dysfunction that did not occur in normal mice [45]. In the current study participants were fit and healthy, which could be why they did not experience endothelial dysfunction at rest. In studies such by Mills et al [7,46], it is unclear how fit participants were, and it is possible that the participants in this study were more fit that those in the studies by Mills et al., [7,46] and thus has some protection against air pollution induced endothelial function at rest. Despite their being no effect at rest, it is still possible to assess the effects during exercise as participants inhaled 4–8 times more particulate matter than during rest.

Literature on how acute exposure to PM during exercise affects vascular function is conflicting. In the current study we found that exercise in DE did not affect FMD which is similar to the findings of Wauters et al., who demonstrated that moderate exercise in DE did not result in vascular dysfunction.[13] In contrast, other studies have found that exercise in a high PM_1_ environment reduced FMD.[11,12] Specifically, Rundell et al. determined that 30 min of exercise in a high PM_1_ environment at a similarly high intensity reduced FMD [12,15] and Cutrufello et al. [11] found that exposure to PM during 26 min of exercise that included a six-minute maximal bout also decreased FMD. In the current study, participants were exposed to a higher PNC (61.60 x 10^4^) than participants in the study by Rundell et al.,[12] (14.4 x 10^4^) and Cutrufello et al., [11] (33.9 x 10^4^ and 34.5 x 10^4^); despite the higher concentration of particles in the current study we did not find a reduction in FMD. It is possible that the opposition in findings is due to methodological differences in how FMD was analysed. In the current study, automated analysis of continuous video recording was performed of video images, as opposed to manual analysis of still images in the prior two studies [11,12,15] Based on the above results, it is challenging to determine how exercise in air pollution containing PM affects vascular endothelial function. One of the major challenges faced when comparing these studies is heterogeneity in the methods of assessing vascular function. For example, Wauters et al. [17] measured microvascular function by assessing the vascular response to sodium nitroprusside and acetylcholine following skin heating, while other studies use FMD, measure forearm blood flow in response to bradykinin, sodium nitroprusside, and acetylcholine, or measure microvascular function at the fingertip. Although the differing assessment techniques and exercise protocols make it challenging to draw conclusions about whether exercise in DE impairs vascular function, the current study does not provide evidence to support this hypothesis.

One limitation within this study is that diesel exhaust chemical composition and particle size vary significantly with engine type, operating conditions, and fuel formations [47]; therefore, the mixture within the current study differs from ambient conditions and other laboratories using DE [24]. Our exposure facility uses a newer engine relative to some older human exposure facilities and likely it produces particulate matter that is less oxidising than older models. Therefore, it is possible that some of our study endpoints would be more altered in a more oxidizing environment. Despite this consideration, DE was chosen as a model air pollution mixture as it contains both gaseous and particulate pollution and represents a mixture similar to that in an urban street canyon with significant heavy goods truck traffic. We also cannot discount that physiological responses will differ with differences in exercise duration, fitness level and/or health status. We chose a 30 min exercise bout to represent a cycle commute [20]; however, we cannot predict how our results would have been different following longer duration exercise. We assessed outcome variables following a 20 min resting period after the cessation of exercise and for up to 2 h post exercise. Therefore, it also cannot be discounted that exposure to DE could have resulted in physiological changes that occurred prior to or after the time that they were assessed. In the present study we assessed endothelial dependent dilation and not endothelial independent dilation; it is possible that in the current study the effects of diesel exhaust on the vasculature could occur independent of the endothelium.

## Conclusion

In healthy recreationally active males, we assessed the endothelial responses to 30 min of rest, low-intensity cycling, and high-intensity cycling with and without DE exposure. Following 30 min of exposure to DE, endothelin-1 was lower compared to FA (2 h post); and following exercise in DE, plasma NOx increased compared to pre-exercise and was significantly higher than FA. Whereas exercise in DE did not affect FMD or blood pressure. The higher plasma NOx during both exercise intensities in DE could increase the oxidative stress potential through the combination of NO with reactive oxygen species.

As DE exposure did not adversely affect endothelial function, the strategy of advising healthy individuals to moderate or avoid exercise during bouts of high pollution was not supported by the findings, for these particular endpoints. Since the observed changes were small, and the exposure concentrations of PM were high, the clinical significance of these findings remains unknown. Despite this relationship, such research may be important to consider in clinical populations where the physiological changes observed in this study could be more significant.

## Supporting information

S1 File Supplementary data file. (XLSX)
